# The ascending aorta of male hypertensive bicuspid aortic valve patients preferentially associated with a cellular aneurysmal phenotype

**DOI:** 10.14814/phy2.15251

**Published:** 2022-04-19

**Authors:** Alexandre Bergeron, Vanessa Hertig, Louis Villeneuve, Vincent Chauvette, Ismail El‐Hamamsy, Angelino Calderone

**Affiliations:** ^1^ Research Center Montreal Heart Institute and Université de Montréal Montreal Quebec Canada; ^2^ Department of Cardiac Surgery Université de Montréal Montreal Quebec Canada; ^3^ Department of Cardiovascular Surgery Icahn School of Medicine at Mount Sinai Mount Sinai Hospital New York New York USA; ^4^ Department of Pharmacology & Physiology Université de Montréal Quebec Montreal Canada

**Keywords:** ascending aorta, BAV, cell cycle inhibitors, hypertension, matrix metalloproteinases, sex

## Abstract

Male sex and hypertension represent risk factors in the progression of an aortic aneurysm. The present study examined the morphological/cellular phenotype of the ascending aorta (AA) of male and female patients diagnosed with a bicuspid aortic valve (BAV) to test the hypothesis that hypertension‐induced remodeling of male BAV patients preferentially recapitulated the expression of a panel of proteins favoring aneurysm formation. The diameter of the AA of hypertensive male (35 ± 6 mm) and female (39 ± 5 mm) BAV patients was comparable to normotensive patients reflecting an early phase of vessel expansion. Morphological/structural remodeling of the medial region of the AA of male normotensive and hypertensive BAV patients were comparable. Protein levels of non‐muscle myosin IIB, the cell cycle inhibitor p27kip1, tumor suppressor p53 and matrix metalloproteinase‐2 and −9 were significantly upregulated in the AA of male hypertensive BAV patients. In female hypertensive BAV patients, collagen content was significantly increased whereas elastin content and medial width of the AA were similar to normotensive BAV patients. In the AA of female hypertensive BAV patients, matrix metalloproteinase‐9 and p27kip1 protein levels were unchanged whereas p53 and matrix metalloproteinase‐2 protein expression was significantly reduced. Nestin protein levels were diminished in the AA of male and female hypertensive BAV patients. Thus, sexual dimorphic remodeling of the AA was prevalent in hypertensive BAV patients. Moreover, during the early phase of vessel expansion, the AA of male hypertensive BAV patients was preferentially associated with the upregulation of a panel of proteins linked to progressive dilatation and potential aneurysm formation.

## Graphical abstract

Sexual dimorphic remodeling of the ascending aorta was prevalent in hypertensive bicuspid aortic valve patients and the cellular phenotype reported in male hypertensive patients preferentially recapitulated a pattern of expression favoring further vessel expansion and potential aneurysm formation.

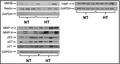

## 1 INTRODUCTION

Male sex and hypertension represent seminal risk factors in the progression of thoracic and abdominal aneurysms (Bossone & Eagle, 2021). Among the various etiologies associated with aneurysm formation, patients with a congenital bicuspid valve are susceptible to thoracic aortic expansion (Bossone & Eagle, 2021; Foffa et al., 2012; Mazine et al., 2018; Michelena et al., 2011; Verma & Siu, 2014). Bicuspid aortic valve (BAV) represents the most common cardiac malformation affecting 1%–2% of the population leading to aortic regurgitation and/or aortic stenosis (Bossone & Eagle, 2021; Foffa et al., 2012; Mazine et al., 2018; Michelena et al., 2011; Verma & Siu, 2014). Moreover, BAV patients were further predisposed to an increased risk of aortic root dilatation and/or aneurysm formation (Bossone & Eagle, 2021; Foffa et al., 2012; Mazine et al., 2018; Michelena et al., 2011; Verma & Siu, 2014). Defects in neural crest stem cell function/migration during the formation of the aortic valve and the ventricular outflow tract may in part contribute to the increased prevalence of aortic aneurysm in this population (Verma & Siu, 2014). Furthermore, increased shear stress in the ascending aorta, caused by the turbulent flow originating from the abnormal valve may alone or in conjunction with genetic defects further exacerbate aneurysm formation (Verma & Siu, 2014). Among a population of male and female BAV patients, Michelena et al. identified aortic stenosis as an independent risk factor of aneurysm formation (Foffa et al., 2012). The study by Foffa et al. further revealed that hypertension and the angiotensin converting enzyme deletion gene had a synergistic impact on the risk of aneurysm formation in BAV patients (Chan & Rabkin, 2014). Consistent with the latter premise, the incidence of hypertension was significantly increased in patients with a thoracic aortic aneurysm and antihypertensive treatment was considered essential to reduce wall stress thereby limiting the risk of subsequent aortic dissection (Olsson et al., 2006). In addition to hypertension, sex represents an additional variable influencing thoracic aortic aneurysm formation as the disease was more prevalent in males albeit the prognosis was significantly worse in females (Kong et al., 2020; Nienaber et al., 2004). In the study by Neinaber et al., the presence of a thoracic aortic aneurysm in women was associated with a greater risk of dissection/rupture as compared to men (Nienaber et al., 2004).

Salient morphological and cellular features of an aneurysmal vessel include medial degeneration secondary to structural dysregulation characterized by elastin fragmentation and subsequent content loss, collagen degradation and the concomitant inability of vascular smooth muscle cells to re‐enter the cell cycle (Allaire et al., 1998; Cui et al., 2017; Sakalihasan et al., 2018; Shen & LeMaire, 2017; Wågsäter et al., 2013). Structural dysregulation of the medial region was attributed in part to the increased expression of matrix metalloproteinases, reduced protein levels of the tissue inhibitor of matrix metalloproteinases and/or impaired collagen cross linking secondary to a decreased expression and/or activity of lysyl oxidase (Allaire et al., 1998; Cui et al., 2017; Sakalihasan et al., 2018; Shen & LeMaire, 2017; Wågsäter et al., 2013). Moreover, vascular smooth muscle cells that failed to re‐enter the cell cycle and/or enter a state of cellular senescence represent an important local source of matrix metalloproteinases (Monk & George, 2015; You et al., 2019; Zang et al., 2017). The absence of cell cycle re‐entry and/or cellular senescence of vascular smooth muscle cells share in part a common phenotype characterized by the upregulation of cyclin‐dependent kinase inhibitors p21^cip1^, p27^kip1^ and the tumor suppressor p53 (Chen et al., 2016; Kuang et al., 2016; Terzi et al., 2016). Finally, several studies have reported that filament proteins non‐muscle myosin IIB and nestin drive cytokinesis of normal and tumor cells and influence matrix metalloproteinase expression (Kim et al., 2018; Lee et al., 2014; Sharma et al., 2019; Takeda et al., 2003; Tardif et al., 2015). Despite their established biological roles, regulation of either filament protein in the ascending aorta of male and female BAV patients during vessel expansion or in the presence of hypertension remains undefined. Thus, the present study examined the morphological/cellular phenotype of the ascending aorta of normotensive and hypertensive male and female BAV patients during the early phase of vessel expansion to test the hypothesis that male sex and underlying hypertension preferentially recapitulated the expression of a panel of proteins favoring progressive vessel expansion and potential aneurysm formation.

## 2 MATERIAL AND METHODS

### 2.1 Ethical approval

The use of human tissue was approved by the Montreal Heart Institute Scientific Ethics Committee on Human Research. All methods were performed in accordance with the relevant guidelines and regulations stipulated by the Montreal Heart Institute Scientific Ethics Committee on Human Research. The guidelines and regulations were formulated by the Canadian Tri‐Council (Canadian Institutes of Health Research, Natural Sciences and Engineering Research Council of Canada Social Sciences and Humanities Research Council) policy of Ethical Conduct for Research Involving Humans. These guidelines and regulations were derived from various organizations including those indicated in the Helsinki Declaration on human research. Informed consent was obtained from each patient that participated in the study prior to surgery.

### 2.2 Tissue collection and preparation

Normotensive and hypertensive male and female bicuspid aortic valve (BAV) patients undergoing the Ross procedure at the Montreal Heart Institute were selectively examined in the present study (Table 1). Transthoracic and transesophageal echocardiograms were used to determine the phenotype and pathology of the aortic valve, the absolute diameter of the aortic annulus and ascending aorta.(Devereux et al., 1997; Emmott et al., 2021) All echocardiograms were performed and analyzed by American board‐certified anesthesiologists. In the male BAV cohort, 12 normotensive and hypertensive male BAV patients were associated with right‐left coronary cusp fusion whereas four normotensive and three hypertensive patients had a right‐non coronary artery cusp fusion. In the female BAV cohort, six normotensive and hypertensive patients were associated with right‐left coronary cusp fusion whereas three normotensive and three hypertensive patients had a right‐non coronary artery cusp fusion. A history of hypertension (blood pressure > 140/90 mmHg) was established by the primary physician, confirmed by a cardiologist at the Montreal Heart Institute and patients were treated with antihypertensive medication (e.g. angiotensin converting enzyme inhibitors, AT‐1 or calcium channel blockers). The inner curvature of the ascending aorta harvested during the Ross procedure was used for immunohistochemistry, immunofluorescence and Western blot experiments. Lastly, for the male BAV cohort, the number of normotensive and hypertensive patients used in the present study was greater than females because the tissue procured from numerous male patients after the Ross procedure surgery was not sufficient to concomitantly perform immunohistochemistry and western blot experiments.

**TABLE 1:** Demographics and clinical characteristics of hypertensive patients

Variable	Male			Female		
	NT	HT	*p*‐value	NT	HT	*p*‐value
	*N* = 16	*N* = 15		*N* = 9	*N* = 9	
Age, years	57 ± 6	56 ± 4	ns	52 ± 6	53 ± 5	ns
Aortic annulus diameter (mm)	25 ± 2	26 ± 2	ns	23 ± 1	24 ± 2	ns
Ascending Aorta (AA) outer diameter (mm)	34 ± 5	35 ± 6	ns	38 ± 7	39 ± 5	ns
Height (cm)	172 ± 9	173 ± 7	ns	159 ± 6	158 ± 4	ns
Weight (kg)	82 ± 16	92 ± 20	ns	80 ± 21	74 ± 17	ns
BSA (m^2^)	1.9 ± 0.3	2.0 ± 0.2	ns	1.8 ± 0.2	1.8 ± 0.2	ns
AA Height Index (cm/m)	2.0 ± 0.3	2.0 ± 0.3	ns	2.4 ± 0.4	2.5 ± 0.3	ns
AA Size Index (cm/m^2^)	1.8 ± 0.3	1.7 ± 0.2	ns	2.1 ± 0.4	2.3 ± 0.4	ns
Aortic root diameter (mm)	34 ± 4	34 ± 4	ns	30 ± 3	34 ± 5	ns
Not available	3 (18.8)	3 (20.0)		2 (22.2)	2 (22.2)	
*Ascending aorta diameter*			ns			ns
<38 mm	11 (68.8)	9 (60.0)		4 (44.4)	3 (33.3)	
38–44 mm	3 (18.8)	5 (33.3)		3 (33.3)	5 (55.6)	
45–50 mm	1 (6.3)	0 (0)		1 (11.1)	0 (0)	
>50 mm	0 (0)	0 (0)		0 (0)	0 (0)	
Not available	1 (6.3)	1 (6.7)		1 (11.1)	1 (11.1)	
*Ascending aorta size index*			ns			ns
<2.00 cm/m^2^	11 (68.8)	12 (80.0)		3 (33.3)	2 (22.2)	
2.00–2.74 cm/m^2^	4 (25.0)	2 (13.3)		5 (55.6)	6 (66.7)	
2.75–4.24 cm/m^2^	0 (0)	0 (0)		0 (0)	0 (0)	
≥4.25 cm/m^2^	0 (0)	0 (0)		0 (0)	0 (0)	
Not available	1 (6.3)	1 (6.7)		1 (11.1)	1 (11.1)	
*Surgical indication*			ns			ns
Aortic stenosis	13 (81.3)	11 (73.3)		9 (100)	9 (100)	
Aortic regurgitation	0 (0)	0 (0)		0 (0)	0 (0)	
Mixed disease	3 (18.8)	4 (26.7)		0 (0)	0 (0)	
*Medical history*						
Hypertension	0 (0)	15 (100)	<0.001	0 (0)	15 (100)	<0.001
Pulmonary hypertension	0 (0)	0 (0)	ns	0 (0)	1 (11.1)	ns
Previous CAD	2 (12.5)	3 (20,0)	ns	0 (0)	0 (0)	ns
Diabetes	1 (6.3)	1 (6.7)	ns	0 (0)	2 (22.2)	ns
Obesity (BMI >30)	7 (43.8)	10 (66.7)	ns	4 (44.4)	2 (22.2)	ns
*Smoking history*						ns
No	13 (81.3)	11 (66.7)	ns	8 (88.9)	7 (77.8)	
Yes	3 (18.8)	4 (26.7)		1 (11.1)	2 (22.2)	
*Valve*			ns			ns
BAV	16 (100)	15 (100)		9 (100)	9 (100)	

Note

Data presented as Mean ± SD or *n* (%). Hypertension was defined with a blood pressure >140/90 mmHg and pulmonary hypertension >30 mmHg. The ascending aorta (AA) outer diameter was indexed to height and body surface area (BSA). *p* values comparing normotensive (NT) and hypertensive (HT) patients of each group were determined by *t*‐test, *χ* ^2^ test or Fisher's exact test. *p* < 0.05 was considered statistically significant.

### 2.3 Histology

Vascular tissue sections (6–8 μm thick) of the ascending aorta were stained using Masson's trichrome and Movat's pentachrome protocols to assess collagen and elastin levels, respectively (Emmott et al., 2021; Tardif et al., 2015). Within each tissue, five arbitrary regions (area; ~0,31 mm^2^) were examined at 20× magnification to avoid biased sampling of the immunohistochemical analysis The surface area of blue‐stained collagen and black‐stained elastin fibers were measured using Image Pro Software to assess the average % of collagen and elastin levels. Lastly, the medial width of the ascending aorta was determined in Masson trichrome stained tissue.

### 2.4 *Western* *blot*

Protein lysates were prepared from the ascending aorta of BAV patients and subjected to Western blot analysis (Emmott et al., 2021; Tardif et al., 2015). Antibodies used were mouse monoclonal anti‐calponin (1:5000; Santa Cruz Biotechnology); mouse monoclonal anti‐smooth muscle 22α (1:5000; Santa Cruz Biotechnology), rabbit polyclonal anti‐non‐muscle myosin IIB (1:5000; Cell Signaling Technologies), rabbit polyclonal anti‐matrix metalloproteinase‐2 (MMP‐2; recognizes proenzyme/cleaved enzyme, 1:5000; Cell Signaling Technologies), mouse monoclonal anti‐matrix metalloproteinase‐9 (MMP‐9, recognizes proenzyme, 1:1000; Thermo Fisher Scientific), tissue inhibitor of metalloproteinase‐1 (1:100; Santa Cruz Biotechnology), chicken polyclonal anti‐nestin (1:1000; Abcam), rabbit polyclonal anti‐p27^kip1^ (1:2000; Santa Cruz Biotechnology), mouse monoclonal anti‐p21^cip1^ (1:500; Santa Cruz Biotechnology), mouse monoclonal anti‐p53 (1:500; Santa Cruz Biotechnology), and a mouse monoclonal anti‐glyceraldehyde phosphate dehydrogenase (GAPDH, 1:50000; Ambion). Thereafter, the appropriate secondary antibody‐conjugated to horseradish peroxidase (1:20,000; Jackson Immunoresearch) was added and bands visualized utilizing the ECL detection kit (Perkin Elmer). Films were scanned with Image J software^®^ and the target protein signal was depicted as arbitrary light units normalized to GAPDH (Emmott et al., 2021; Tardif et al., 2015).

### 2.5 Immunofluorescence

Ascending aortic tissue sections (6–8 μm thick) were fixed and subjected to immunofluorescence (Emmott et al., 2021; Tardif et al., 2015). Tissues were incubated with chicken polyclonal anti‐nestin (1:100; Abcam) and rabbit monoclonal anti‐non‐muscle myosin IIB (1:100; Abcam). Secondary antibodies employed were goat anti‐chicken IgG conjugated to Alexa‐555 (1:600; Life Technologies) and goat anti‐rabbit IgG conjugated to Alexa‐647 (1:600; Life Technologies; Emmott et al., 2021; Tardif et al., 2015). The nucleus was identified with 4′,6′‐diamidino‐2‐phenylindole (DAPI, 1.5 μM; emission wavelength, 460 nm; Sigma‐Aldrich) staining. Non‐specific staining was determined following the addition of the conjugated secondary antibody alone.

### 2.6 *Statistical* *analyses*

In Table 1, the data are presented as mean ± SD or *n* (%) and represents patients in which the ascending aorta was used for morphological and/or cellular analysis. *p* values comparing normotensive and hypertensive patients were determined by *t*‐test, *χ* ^2^ test or Fisher's exact test and a *p* < 0.05 was considered statistically significant. Western blot and morphological analysis were determined with an unpaired *t*‐test and value of *p* < 0.05 considered statistically significant (GraphPad Prism 8).

## 3 RESULTS

### 3.1 Classification of normotensive and hypertensive male patients with a bicuspid aortic valve

Male normotensive and hypertensive patients were predominantly diagnosed with aortic stenosis whereas a modest number presented with mixed disease (e.g., stenosis and regurgitation; Table 1). Male normotensive and hypertensive BAV populations consisted of patients of similar age and the absolute average diameter of the preoperative aortic annulus, the aortic root and the ascending aorta were comparable (Table 1). Normalizing the ascending aortic diameter to height or body surface area (BSA) of each patient further revealed no significant difference between male normotensive and hypertensive BAV patients (Table 1). In addition, the majority of normotensive and hypertensive male BAV patients were associated with an aortic index <2.00 cm/m^2^ (Table 1). Based on the study by Davies et al., the risk of aortic rupture was reported to be less than 4% in patients with an aortic index <2.74 cm/m^2^ (Davies et al., 2006). Consistent with the latter premise, the absolute value of the diameter of the ascending aorta of male normotensive (34 ± 5 mm) and hypertensive (35 ± 6 mm) BAV patients examined at the time of surgery revealed an early stage of vessel expansion as the absolute diameter of the ascending aorta of normal males older than 40 years old was 27–30 mm (Table 1; Campens et al., 2014; Fatehi Hassanabad et al., 2019; Kallenbach et al., 2013; Vriz et al., 2014). Moreover, previous studies have reported that surgical intervention is recommended when the diameter of the ascending aorta is greater than 50 mm (Campens et al., 2014; Fatehi Hassanabad et al., 2019; Kallenbach et al., 2013; Vriz et al., 2014).

### 3.2 Morphological and cellular remodeling of the ascending aorta of male hypertensive BAV patients

**FIGURE 1:**
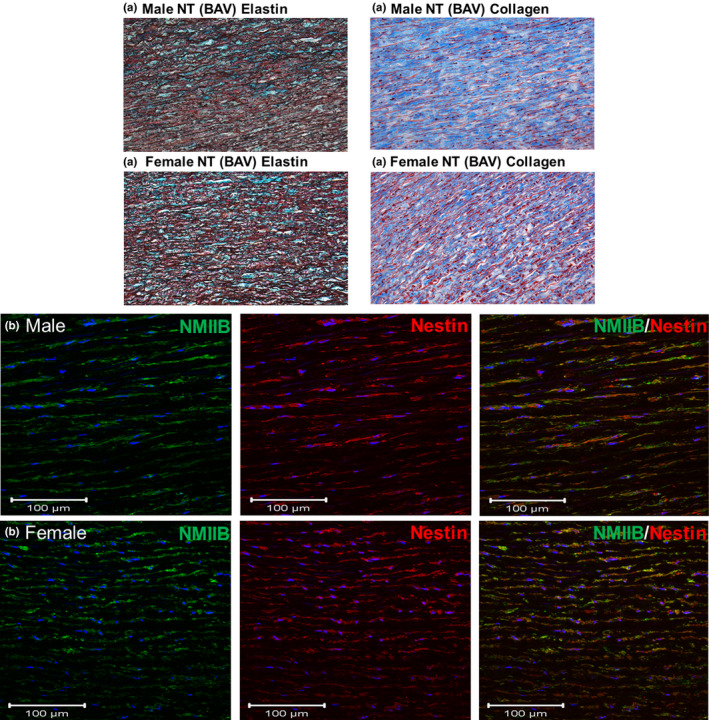
Morphological and cellular phenotype of the ascending aorta of male and female normotensive bicuspid aortic valve (BAV) patients diagnosed with aortic stenosis. (a) Immunohistochemical data depicting collagen (blue hue) and elastin (black hue fibres) staining of the medial region of the ascending aorta of male normotensive (NT) and female (NT) normotensive BAV patients. (b) Nestin and non‐muscle myosin IIB (NMIIB) immunofluorescence co‐staining of vascular smooth muscle cells was identified in the medial region of the ascending aorta of male and female normotensive BAV patients. The nucleus identified with DAPI staining (blue fluorescence)

**FIGURE 2:**
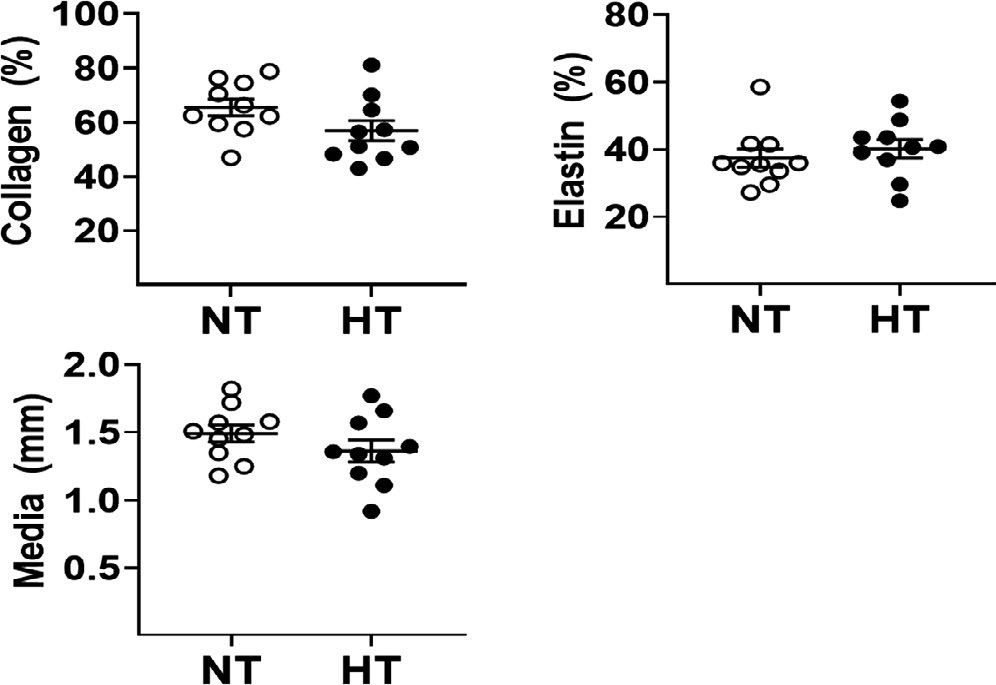
Morphology of the ascending aorta of male normotensive and hypertensive bicuspid aortic valve (BAV) patients diagnosed with aortic stenosis. Collagen and elastin content in the medial region and the medial width of the ascending aorta of male hypertensive BAV patients diagnosed with aortic stenosis were similar to male normotensive BAV patients

Immunohistochemistry revealed blue‐stained collagen and black‐stained elastin fibers in the medial region of the ascending aorta of male BAV patients (Figure 1a). In male hypertensive BAV patients (*n* = 10), medial thickness of the ascending aorta was similar to normotensive BAV patients (*n* = 10). Furthermore, collagen and elastin density in the medial region of the ascending aorta were comparable between male normotensive and hypertensive BAV patients (Figure 2). Thus, at the time of surgery, the structural/morphological phenotype of the ascending aorta of male hypertensive BAV patients was comparable to normotensive BAV patients.

**FIGURE 3:**
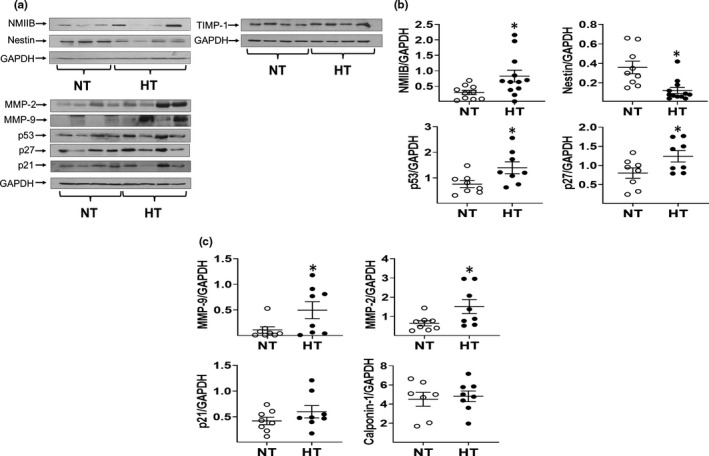
Cellular remodeling of the ascending aorta of male normotensive and hypertensive bicuspid aortic valve (BAV) Patients diagnosed with Aortic Stenosis. (a) Protein expression of non‐muscle myosin IIB (NMIIB), nestin, matrix metalloproteinase‐2 (MMP‐2), matrix metalloproteinase‐9 (MMP‐9), tissue inhibitor of matrix metalloproteinase‐1 (TIMP‐1), p53, p27^kip1^, and p21^cip1^ in the aorta of male normotensive (NT) and hypertensive (HT) BAV patients. (b, c) Semi‐quantitative analysis of the Western blot data revealed an upregulation of NMIIB, MMP‐2, MMP‐9, p27^kip1^ and p53, whereas nestin protein levels were significantly reduced in the ascending aorta of male hypertensive (HT) patients compared to male normotensive (NT) BAV patients. Protein expression was normalized to GAPDH protein levels and (*) denotes *p* < 0.05 versus male normotensive BAV patients

In the medial region of the ascending aorta of male normotensive BAV patients, immunofluorescence revealed that vascular smooth muscle cells co‐expressed the filament proteins non‐muscle myosin IIB and nestin (Figure 1b). In the presence of underlying arterial hypertension, protein levels of non‐muscle myosin IIB were significantly upregulated in the ascending aorta of male hypertensive BAV patients compared to male normotensive BAV patients (Figure 3a,b). By contrast, expression of the intermediate filament protein nestin was significantly reduced in the ascending aorta of male hypertensive BAV patients (Figure 3a,b). To indirectly assess the proliferative state of aortic vascular smooth muscle cells, protein expression of cyclin‐dependent kinase inhibitors p21^cip1^ and p27^kip1^, and the tumor suppressor p53 proliferating were examined. In the ascending aorta of male BAV hypertensive patients, p27^kip1^ and p53 proteins levels were markedly upregulated in a subpopulation of patients that translated to a significant difference as compared to normotensive patients (Figure 3a,b). By contrast, p21^cip1^ protein expression in the ascending of aorta of male hypertensive patients was comparable to male normotensive BAV patients (Figure 3a,c). Likewise, matrix metalloproteinase‐2 and ‐9 protein levels were robustly increased in the ascending aorta of a subpopulation of male hypertensive BAV patients that subsequently translated to an overall significant difference as compared to normotensive BAV patients (Figure 3a,c). By contrast, protein expression of the inhibitor of metalloproteinase‐1 expression (Normotensive 1.10 ± 0.15 versus Hypertensive 1.45 ± 0.27; *n* = 7 for each group; normalized to GAPDH; Figure 3a) was similar between male normotensive and hypertensive patients. Lastly, smooth muscle 22α (normotensive 1.4 ± 0.1 vs. hypertensive 1.7 ± 0.2; *n* = 8 for each group; normalized to GAPDH) and calponin‐1 protein levels (Figure 3c) were similar between normotensive and hypertensive groups. These data reveal that underlying arterial hypertension led to the upregulation of a panel of proteins in the ascending aorta of male BAV patients previously reported to promote progressive vessel expansion and subsequent aneurysm formation.

### 3.3 Classification of normotensive and hypertensive female patients with a bicuspid aortic valve

Female normotensive and hypertensive patients were exclusively associated aortic stenosis, were of similar age and the absolute average diameter of the preoperative aortic annulus, the aortic root and the ascending aorta were comparable (Table 1). Furthermore, normalizing the ascending aortic diameter to height or BSA of each patient revealed no significant difference between normotensive and hypertensive patients (Table 1; Campens et al., 2014; Davies et al., 2006; Fatehi Hassanabad et al., 2019; Kallenbach et al., 2013; Vriz et al., 2014). In addition, the majority of normotensive and hypertensive female BAV patients were associated with an aortic index <2.74 cm/m^2^ (Table 1). Thus, based on these data and as identified in the male BAV populations, the risk of aortic rupture in female BAV patients was less than 4% (Davies et al., 2006). Consistent with the latter premise, the absolute value of the diameter of the ascending aorta of female normotensive (38 ± 7 mm) and hypertensive (39 ± 5 mm) BAV patients examined at the time of surgery revealed an early stage of vessel expansion as the absolute diameter of the ascending aorta of normal females older than 40 years old was 27–30 mm (Table 1; Campens et al., 2014; Fatehi Hassanabad et al., 2019; Kallenbach et al., 2013; Vriz et al., 2014).

### 3.4 Morphological and cellular remodeling of the ascending aorta of female hypertensive BAV patients

**FIGURE 4:**
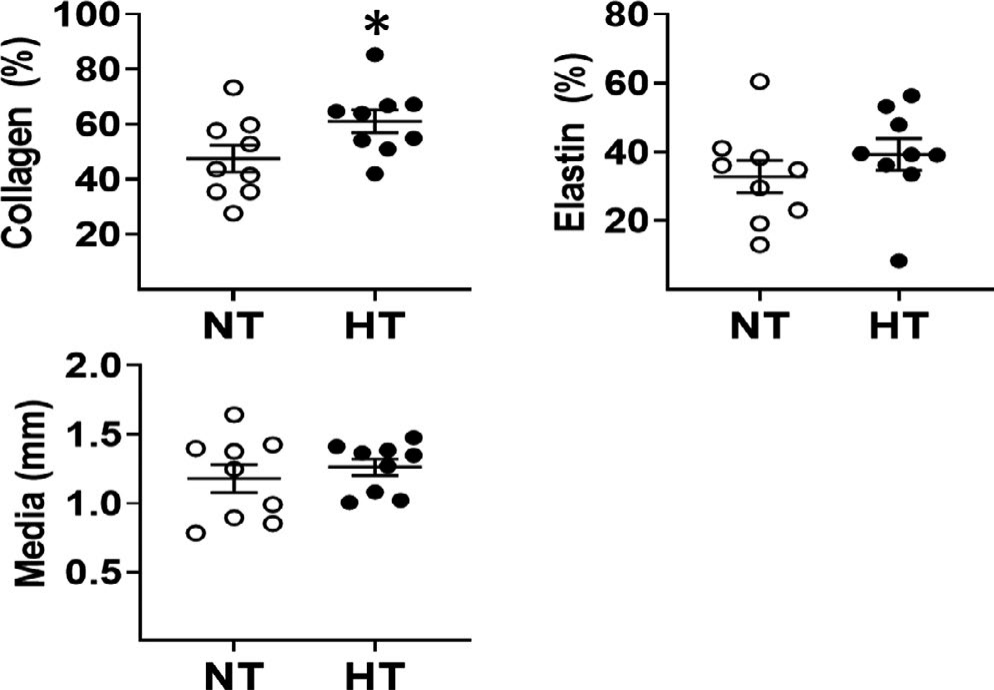
Morphology of the ascending aorta of female normotensive and hypertensive bicuspid aortic valve (BAV) patients diagnosed with aortic stenosis. The medial width and elastin content were unchanged whereas collagen content was increased in the ascending aorta of female hypertensive (HT) BAV patients (*n* = 9) compared to female normotensive (NT) BAV patients (*n* = 9). (*) denotes *p* < 0.05 versus female normotensive BAV patients

Collagen and elastin fiber staining of the medial region of the ascending aorta of normotensive female BAV patients was depicted in Figure 1a. Elastin density in the medial region and the medial width of the ascending aorta of female normotensive BAV patients (*n* = 9) were comparable to female hypertensive BAV patients (*n* = 9; Figure 4). By contrast, collagen density in the medial region of the ascending aorta of female hypertensive BAV patients was significantly greater compared to female normotensive BAV patients (Figure 4). These data reveal that at the time of surgery, the selective upregulation of collagen content distinguished the structural/morphological phenotype of the ascending aorta of female normotensive and hypertensive BAV patients.

**FIGURE 5:**
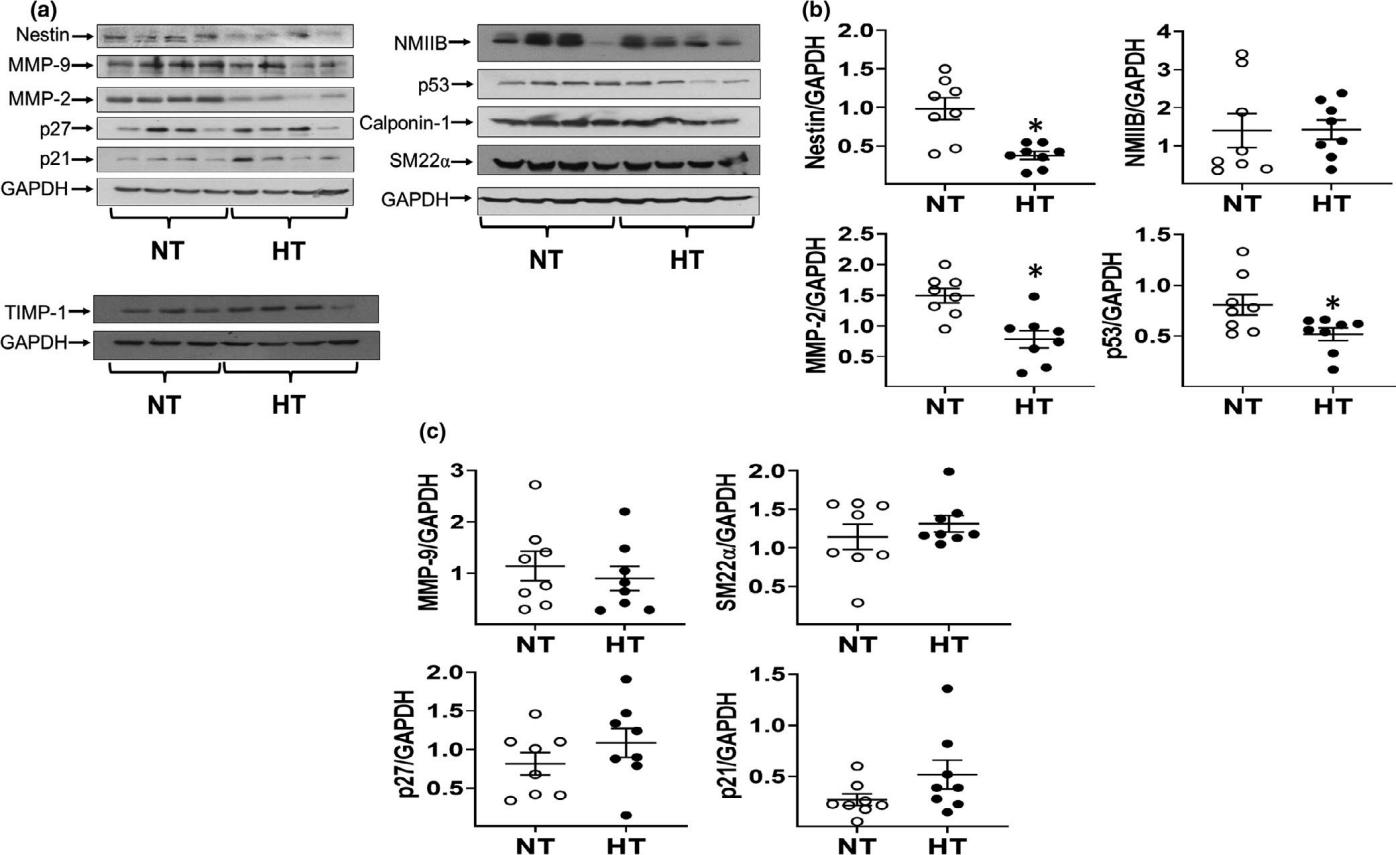
Cellular remodeling of the ascending aorta of female normotensive and hypertensive bicuspid aortic valve (BAV) patients diagnosed with aortic stenosis. (a) Protein expression of nestin, matrix metalloproteinase‐2 (MMP‐2), matrix metalloproteinase‐9 (MMP‐9), tissue inhibitor of matrix metalloproteinase‐1 (TIMP‐1), p27^kip1^, and p21^cip1^, non‐muscle myosin IIB (NMIIB), p53, smooth muscle 22α (SM22α) and calponin‐1 in the ascending aorta of female normotensive (NT) (*n* = 7–8) and hypertensive (HT) BAV patients (*n* = 8). (b, c) Semi‐quantitative analysis of the Western blot data revealed that protein levels of nestin, MMP‐2, and p53 were significantly reduced in the ascending aorta of female hypertensive (HT) BAV patients as compared to normotensive (NT) BAV patients. Protein expression was normalized to GAPDH protein levels and (*) denotes *p* < 0.05 versus female normotensive patients

In the medial region of the ascending aorta of female normotensive BAV patients, immunofluorescence revealed that vascular smooth muscle cells co‐expressed the filament proteins non‐muscle myosin IIB and nestin (Figure 1b). Analogous to that observed in male hypertensive BAV patients, nestin protein levels were significantly reduced in the ascending aorta of female hypertensive BAV patients compared to normotensive BAV patients (Figure 5a,b). In contrast to male hypertensive BAV patients, matrix metalloproteinase‐2 and p53 expression were significantly reduced in the ascending aorta of female hypertensive BAV patients (Figure 5a–c). Non‐muscle myosin IIB, p21^cip1^, p27^kip1^, matrix metalloproteinase‐9 and tissue inhibitor of metalloproteinase‐1 (Normotensive 1.03 ± 0.15 vs. Hypertensive 1.22 ± 0.19; *n* = 7 for each group; normalized to GAPDH) protein levels were unchanged in the ascending aorta of female hypertensive BAV patients compared to normotensive BAV patients (Figure 5a–c). Lastly, calponin‐1 (Normotensive 1.34 ± 0.11 vs. Hypertensive 1.10 ± 0.10; *n* = 8 for each group; normalized to GAPDH; Figure 5a) and smooth muscle 22α protein levels in the ascending aorta of female normotensive and hypertensive patients were likewise similar (Figure 5a,c). Thus, in the ascending aorta of female BAV patients, underlying arterial hypertension failed to recapitulate the morphological/cellular phenotype observed in male hypertensive BAV patients.

## 4 DISCUSSION

Hypertension and sex represent independent risk factors of aortic aneurysm formation and an analogous clinical observation was identified in patients diagnosed with BAV (Bossone & Eagle, 2021; Chan & Rabkin, 2014; Foffa et al., 2012; Mazine et al., 2018; Michelena et al., 2011; Verma & Siu, 2014). Therefore, the present study examined the morphological/cellular features of the ascending aorta of normotensive and hypertensive male and female BAV patients during the early phase of vessel expansion to test the hypothesis that arterial hypertension selectively recapitulated the expression of a panel of proteins in male BAV patients that increased the risk of vessel expansion and potential aneurysm formation. Previous studies have reported that the absolute diameter of the ascending aorta of normal males and females older than 40 years old was 27–30 mm (Campens et al., 2014; Vriz et al., 2014). Furthermore, surgical intervention is recommended when the diameter of the ascending aorta was greater than 50 mm (Fatehi Hassanabad et al., 2019; Kallenbach et al., 2013). In the present study, the average absolute diameter of the ascending aorta (~34 mm) and/or normalized to height or BSA of male BAV patients ~57 years old supports the premise that the vessel was in an early stage of expansion (Campens et al., 2014; Davies et al., 2006; Fatehi Hassanabad et al., 2019; Kallenbach et al., 2013; Vriz et al., 2014). Morphologically, collagen and elastin density in the medial region and the medial width of the ascending aorta of male hypertensive BAV patients was comparable to normotensive patients supporting the absence of a structural aneurysmal phenotype. Despite a similar morphological/structural phenotype, protein expression of the tumor suppressor p53 and cyclin‐dependent kinase inhibitor p27^kip1^ were upregulated in the ascending aorta of male hypertensive BAV patients. These data suggest that cell cycle re‐entry of vascular smooth muscle cells in the ascending aorta of male hypertensive BAV patients may have been in part compromised and/or in the process of acquiring a senescent phenotype (Chen et al., 2016; Kuang et al., 2016; Monk & George, 2015; Terzi et al., 2016; You et al., 2019; Zang et al., 2017). Furthermore, matrix metalloproteinase‐2 and ‐9 protein levels were significantly elevated in the ascending aorta of male BAV patients compared to normotensive BAV patients (Cui et al., 2017; Sakalihasan et al., 2018; Shen & LeMaire, 2017). Previous studies have reported that increased matrix metalloproteinase expression facilitated an aneurysmal phenotype characterized by collagen degradation and/or elastin fragmentation (Cui et al., 2017; Sakalihasan et al., 2018; Shen & LeMaire, 2017). However, at the time of surgery, collagen and elastin content in the ascending aorta of male hypertensive BAV patients was similar to normotensive male patients. Therefore, despite elevated matrix metalloproteinase‐2 and ‐9 protein levels, the comparable morphological/structural phenotype of the ascending aorta of male normotensive and hypertensive BAV patients at an early stage of vessel expansion may be attributed in part to the continued expression of tissue inhibitors of matrix metalloproteinases (Allaire et al., 1998). Indeed, expression of the tissue inhibitor of metalloproteinase‐1 in the ascending aorta of male hypertensive BAV patients was similar to that observed in normotensive BAV patients. The latter data suggest that persistent expression of tissue inhibitor of metalloproteinase‐1 during the early stage of vessel expansion may prevent and/or delay the deleterious impact of matrix metalloproteinases on the structural integrity of the ascending aorta of hypertensive male BAV patients. Consistent with the latter premise, Corbit and colleagues reported that a reduced copy number of the tissue inhibitor of metalloproteinase‐1 gene in BAV patients diagnosed with Turner Syndrome had a greater risk of thoracic aortic aneurysm formation (Corbitt et al., 2018).

Clinical studies have revealed that women are less likely to develop a thoracic aortic aneurysm as compared to age‐matched males (Bossone & Eagle, 2021; Kong et al., 2020; Nienaber et al., 2004). Despite a sex‐related protection to aneurysm formation, women that develop an aneurysm were associated with worse prognosis (Bossone & Eagle, 2021; Kong et al., 2020; Nienaber et al., 2004). The presence of a significant thoracic aortic aneurysm in women was associated with a greater risk of dissection/rupture (Kong et al., 2020). A recent study reported that the greater risk of aortic rupture of a thoracic aneurysm was selectively linked to an underlying degenerative cause as the growth rate of the aneurysm in females was greater compared to age‐matched men (Cheung et al., 2017). By contrast, the latter paradigm was not prevalent in females with a thoracic aortic aneurysm linked to a genetic disorder (Cheung et al., 2017). Akin to the male BAV cohort, the average absolute diameter of the ascending aorta and/or normalized to height or BSA of female BAV patients ~52 years old supports the premise that the vessel was in an early stage of expansion (Campens et al., 2014; Davies et al., 2006; Fatehi Hassanabad et al., 2019; Kallenbach et al., 2013; Vriz et al., 2014). Morphologically, collagen density was significantly increased in the medial region of the ascending aorta of female hypertensive patients whereas elastin density and medial width were similar to normotensive patients. Therefore, the modest level of vessel expansion was not associated with an aneurysmal structural phenotype as collagen and elastin fiber content was maintained in female hypertensive BAV patients. At the cellular level, matrix metalloproteinase‐9, tissue inhibitor of metalloproteinase‐1, p21^Cip1^ and p27^Kip1^ protein levels in the ascending aorta in female normotensive and hypertensive BAV patients were similar. By contrast, matrix metalloproteinase‐2 and tumor suppressor p53 proteins levels were downregulated in the ascending aorta of female hypertensive BAV patients. Thus, in contrast to male BAV patients, underlying arterial hypertension in female BAV patients was not associated with the increased expression of matrix metalloproteinases. Furthermore, the absence of a change in a panel of cell cycle inhibitors suggests that the capacity of vascular smooth muscle cells to re‐enter the cell cycle may not have been compromised in the ascending aorta of female hypertensive BAV patients. Collectively, these data unequivocally reveal that the female sex did not recapitulate the cellular phenotype observed in the ascending aorta of male BAV patients in the presence of underlying arterial hypertension. Lastly, the average age of natural menopause in Canadian women is 51 and the age of the cohort of female patients examined in the present study was 52‐53 years old (Costanian et al., 2018). The present study did not determine whether female BAV patients were menopausal. Nonetheless, based on the existing literature, it would be reasonable to conclude that female BAV patients examined in the present study were pre‐menopausal and/or in a state of menopause. In this regard, it is tempting to speculate that the associated loss of cardiovascular protection secondary to the decline of circulating ovarian hormones estrogen and progesterone in menopausal women did not translate to the upregulation of a panel of proteins implicated in aneurysmal formation in the ascending aorta of female hypertensive BAV patients (Li et al., 2021; Mulvagh et al., 2022; Wild et al., 2021).

Non‐muscle myosin IIB and the intermediate filament protein nestin expressed in numerous cell types including vascular smooth muscle cells, participate in cytokinesis and migration (Betapudi, 2014; Hertig et al., 2018; Kim et al., 2018; Komatsu & Ikebe, 2014; Lee et al., 2014; Meus et al., 2017; Sharma et al., 2019; Takeda et al., 2003; Tardif et al., 2015). In the presence of arterial hypertension, non‐muscle myosin IIB protein levels were upregulated in the ascending aorta of male BAV patients, whereas expression remained unchanged in female hypertensive BAV patients. A recent study reported that the loss of non‐muscle myosin IIB in mouse lung mesenchymal cells translated to a downregulation of collagen, fibronectin and elastin and the concomitant selective upregulation of matrix metalloproteinase‐2 (Takeda et al., 2003). However, the relationship between non‐muscle myosin IIB and extracellular matrix remodeling identified in mouse lungs did not extend to the ascending aorta of male and female hypertensive BAV patients. Furthermore, in contrast to the sex‐dependent regulation of the filament protein non‐muscle myosin IIB, nestin protein levels were downregulated in the ascending aorta of male and female hypertensive BAV patients. These data were in stark contrast to the reported increased expression of nestin in the rat aorta in response to elevated blood pressure secondary to suprarenal aortic constriction (Sharma et al., 2019). A previous study reported that p53‐dependent signaling suppressed nestin expression in hepatic cancer cells (Tschaharganeh et al., 2014). Indeed, p53 upregulation in the ascending aorta of male hypertensive BAV patients was associated with nestin downregulation whereas the latter relationship was not recapitulated in female hypertensive BAV patients. In addition, nestin downregulation in several melanoma cells lines was associated with the concomitant upregulation of matrix metalloproteinase‐1, ‐3, and ‐9 (Lee et al., 2014). The latter causal relationship was observed in the ascending aorta of male hypertensive BAV patients as metalloproteinase‐2 and ‐9 were upregulated. However, the latter pattern was not recapitulated in the ascending aorta of female hypertensive BAV patients as metalloproteinase‐2 expression was reduced and metalloproteinase‐9 protein levels unchanged. Thus, it is tempting to speculate that sex may have likewise influenced the potential interaction between nestin, p53 and metalloproteinase expression in the ascending aorta of BAV patients. Nonetheless, nestin downregulation represents a novel cellular phenotype distinguishing the ascending aorta of normotensive and hypertensive BAV patients diagnosed with aortic stenosis, regardless the sex. Biologically, the reduced expression of the intermediate filament protein nestin may in part compromise the proliferative and migratory phenotype of vascular smooth muscle cells during progressive expansion of the ascending aorta of hypertensive BAV patients.

## 5 CONCLUSION

The ascending aorta of normotensive and hypertensive male and female BAV patients was used to test the hypothesis that underlying arterial hypertension in male BAV patients selectively recapitulated in part a cellular phenotype implicated in progressive vessel expansion and potential aneurysm formation. First, the reported diameter of the ascending aorta and the absence of a structural aneurysmal phenotype supports the premise that the vessel was in an early stage of expansion in both male and female BAV cohorts (Campens et al., 2014; Davies et al., 2006; Fatehi Hassanabad et al., 2019; Kallenbach et al., 2013; Vriz et al., 2014). Second, despite the absence of morphological/structural dysregulation, the present study identified a sexually dimorphic pattern of cellular remodeling of the ascending aorta of hypertensive BAV patients. The cellular phenotype of the ascending aorta of male hypertensive BAV patients at the time of surgery (e.g., increased expression of cell cycle inhibitors and matrix metalloproteinases) recapitulated in part the pattern identified during aortic aneurysm formation. By contrast, in the ascending aorta of female hypertensive BAV patients, vascular smooth muscle cells did not exhibit a cellular senescent phenotype or associated with increased matrix metalloproteinase expression. Thus, the cellular phenotype of the ascending aorta of male hypertensive BAV patients during an early stage of vessel expansion further supports in part the clinical observation that the presence of arterial hypertension translates to a greater risk of progressive dilatation and possible aortic aneurysm formation. Lastly, the study is associated with several limitations. The paucity of information regarding the history of hypertension and the efficacy of the various pharmacological agents to lower and/or prevent blood pressure rise in male and female BAV patients limits in part interpretation of the data. Moreover, additional studies are required to assess whether the sex‐dependent pattern of remodeling of the ascending aorta reported in the present study was exclusive to hypertensive BAV patients or recapitulated in part in hypertensive patients in the absence of aortic valve disease.

## CONFLICT OF INTEREST

The authors declare no conflict of interest.

## AUTHORS CONTRIBUTION

Alexandre Bergeron, Western blot and immunohistochemistry; Vanessa Hertig, Western blot and immunohistochemistry; Louis Villeneuve, Immunofluorescence; Vincent Chauvette, Analysis of immunohistochemistry data; Ismail El‐Hamamsy, procuring human tissue; Angelino Calderone, design of experiment, writing of the manuscript.
